# Geopolymers—Design, Preparation, and Applications

**DOI:** 10.3390/polym14050853

**Published:** 2022-02-22

**Authors:** Ignazio Blanco, Michelina Catauro

**Affiliations:** 1 Department of Civil Engineering and Architecture and INSTM UdR, University of Catania, V.le A. Doria 6, 95125 Catania, Italy; 2 Department of Engineering, University of Campania “Luigi Vanvitelli”, Via Roma 29, 81031 Aversa, Italy

Concrete is the most commonly used construction material worldwide, and many efforts have been carried out in recent years to improve its functional properties while also trying to increase its sustainability. Concrete’s environmental impact is well known, linked both to the space it occupies in landfills and its highly energy-intensive manufacturing, thus branding the cement industry one of the largest CO_2_ emission sources. For this reason, both the sector industry and academia have devoted their studies to eco-sustainable materials able to maintain the specific properties of cements. Synthetic inorganic polymers, based on aluminosilicate or phosphate, and prepared following a process known as geopolymerization, have been recognized for almost twenty years now as geopolymers. Characterized by chemical and mechanical properties comparable to those of ceramics, geopolymers present great tolerance for high temperatures and do not explode, contrary to the traditional hydraulic cements. In addition, geopolymers are non-toxic, eco-friendly, and sustainable materials considering, as testified by many papers of this Special Issue, that waste can be recycled as raw materials or inert fillers for their preparation, maintaining performances comparable to those of Portland cement.

This Special Issue, which consists of 22 articles (including two review articles) written by research groups of experts in the field, considers recent research on the design, preparation, and characterization of geopolymers.

The first paper, published by Lee and coworkers, aims at developing a sustainable structural material for the construction industry, namely a marble-based geopolymer concrete [1]. The viability of this new geopolymer, prepared by using powder collected from marble mines, was evaluated in terms of physical/mechanical properties, which showed very good performances. According to various experimental tests and a large-scale ready-mixed plant test, it was found that the marble-based geopolymer concrete displayed a good workability and was not easily influenced by temperature changes. Although a marble-based geopolymer concrete may not be used in reinforced concrete when compared with Portland cement, it still shows good potential for further engineering applications. 

Additionally, in the work of Kim et al., the focus is on the physical and chemical properties changes of alkali-activated cements prepared from blast furnace slag and fly ash [2], representing an alternative to conventional materials because they can significantly reduce CO_2_ emissions compared to cement concrete. Alkali activators such as sodium silicate and sodium hydroxide were used in the preparation of alkali-activated cements, whose structures are observed to change via carbonation. In addition, they recorded a simultaneous decrease in the compressive strength, thus opening interesting future scenarios in the field of alkali-activated cements.

In their contribution, Le and colleagues discussed the experimental data as regards the mechanical properties of the hybrid composite thin-plates of the short basalt fibers/carbon textile-reinforced geomortar [3]. The effect of fiber contents and lengths on the prepared geopolymer on its mechanical properties was evaluated. These authors observed a marked reduction in workability by increasing the reinforcement despite a significant improvement in both static and dynamic flexural strength. The obtained results allowed them to conclude that neither the basalt fibers dose levels nor the fiber lengths used yielded a positive effect on the failure manner of textile-reinforced geopolymer. In the meantime, the results showed the anchoring capacity of textile layers in geomortar plays an important role in specimens’ strength. 

In order to evaluate the effects of the reinforcement and their estimation as correctly as possible, Shi et al., developed practical prediction models of splitting tensile strength and reinforcement–concrete bond strength of low-calcium fly ash geopolymer concrete [4]. The presented models overcome the overestimation problems of the strength models of Portland cement concrete, and they can be applied for the estimation of cracking behaviors and for the calculation of the design anchorage length of reinforced geopolymer beams. 

Mohd Basri and coworkers, in coating geopolymer with rice husk ash (RHA), tried to balance the increase in fire retardant properties with the water absorption effects due to the incorporation of RHA [5]. Aiming to identify the key factors on moisture absorption and fire retardant properties for determining the optimum composition for the prepared geopolymers, they used statistical analysis approach and a regression coefficient model. In addition, they carried out a microstructure analysis which showed that the char residue of the geopolymer coating gave rise to a shielding effect for the layers underneath. The same authors, trying to gain more information on the fire-retardant properties of these geopolymers by using thermal and spectroscopic techniques, highlighted their glassy appearance, and how the surface coating changed into a dense geopolymer gel covered with thin needles when fired. The absence of crack formation and a low dehydration rate allowed them to conclude that using RHA in geopolymer formulation can potentially improve fire safety in the construction of residential and commercial buildings [6]. Although the insulation materials possess high fire-retardant characteristics, their mechanical properties are relatively poor, thus the effect of different material behavior, namely brittle and ductile, on the compressive strength properties, and the optimum material formulation that can satisfy both compressive strength and fire-retardant properties, were also studied by these authors. Brittle samples with low Si/Al ratio were high in compressive strength, showing a high degree of geopolymerization, differently than ductile samples for which low compressive strength and low degree of geopolymerization were observed [7].

By stressing the positive environmental impact of the geopolymers, both as a friendly alternative for concrete and utilization of hazardous wastes, Górski et al., focused their study on the recovery of discarded cathode ray tube glass as aggregate for the preparation of metakaolin-based geopolymer [8]. After designing the optimal geopolymer composition and establishing the curing conditions, they carried out flexural and compressive strength tests, which demonstrated that neither the glass content nor the curing regime had a significant influence on the mechanical behavior of the obtained geopolymer. Thus, they concluded that this discarded waste can be considered a useful aggregate for a metakaolin-based geopolymer. 

This research line was also followed by Dal Poggetto et al., who reused glass waste prepared geopolymer at different percentages and grain size of glass to evaluate their antimicrobial activity [9]. As the decrease in the mechanical properties, as a function of glass content, indicated their potential use as non-structural materials, the prepared geopolymers were tested for their effects on the microbial growth of *Escherichia coli* and *Enterococcus faecalis*, showing a very limited and absent inhibition zone for fine and coarse grain size ranges, respectively. The same research group, aiming to reduce the percentage of debris disposal derived from urban solid waste, suggested an upcycling process where glass debris is simply ground without any washing operation and added to an alkali-activated paste. Thus, metakaolin-based geopolymer mortar added with coarsely ground glass waste has been prepared and characterized, showing interesting antibacterial properties [10].

Assuming that the addition of magnetite to concrete improves its mechanical properties and durability in terms of water and chloride ions absorption, Sgarbossa et al., proposed the design and preparation of novel magnetic geopolymers based on matrices with and without inert quartz aggregates, containing metal particles at different concentrations [11]. By using a method of general application, considering the wide range of experimental parameters and modulability of the geopolymers preparation, together with the different types and amounts of magnetic particles, they obtained composites with a compressive strength higher than those reported for high weight concretes containing a similar magnetite amount. Furthermore, the electrical resistance is mainly controlled by the matrix’s chemical composition, thus their study showed as it can be used to evaluate the geopolymerization degree.

With a view towards greater sustainability, Lancelotti et al., proposed an alternative cementitious binder based on industrial side streams and characterized by a low carbon footprint [12]. A novel approach was carried out by these authors, by means of chemical measurements instead of the usual mechanical approach, to gain information about consolidation degree in the alkali-activated composites. In particular, they focused on the presence of reinforcement and its effect on the chemistry of polycondensation. For this purpose, cellulose (organic nature) and basalt fiber (inorganic nature) were chosen, showing a different behavior in the alkaline media that was used to activate the slag fine powders. They observed that the presence of the fibers does not favor nor hinder the geopolymerization process, even if an increase in the ionic conductivity in samples containing fibers leads to a lower consolidation. 

Continuing to follow the path of reducing the environmental impact of cementitious formulations, Algaifi and coworkers proposed alkali-activated mortar (AAM) made with binary binders involving fly ash (FA) and granulated blast-furnace slag (GBFS) as well as bottle glass waste nano-silica powder (BGWNP) [13]. By using optimization modelling, they proposed various combinations among the different components taken into consideration, showing that the combination of binary binders (FA and GBFS) and BGWNP increased AAM’s strength compared to that of the control mixture for all scenarios, thus demonstrating not only environmental benefits, but also strength properties enhancement.

Additionally, Wang et al. were interested in the use of concrete reinforcements based on waste materials. In particular, they turned their attention to the huge amounts of waste ceramic tiles and blast furnace slag (BFS) produced by the ceramic tile factories and the iron smelting industry [14]. They mixed waste ceramic powder (WCP) into BFS paste and mortar activated by sodium silicate and sodium hydroxide to study its effect on the performance of the obtained samples. Their results showed the presence of waste ceramic powder lead to a resistance enhancement with respect to the acid and the increase in the chloride ions diffusion coefficient. Finally, the mixture containing ceramic waste presented a better fluidity of the alkali-activated paste.

Quiatchon and coworkers, aiming to increase the low utilization rate of the coal fly ash produced in the Philippines, proposed its use to prepare fly ash-based geopolymer for construction, thus also reaching the goal of lower greenhouse-gas emissions. They provided an impressive amount of specimens in order to optimize the geopolymer components for the replacement of Portland Cement for in situ applications [15]. The engineering properties chosen as the optimization responses, namely the unconfined compressive strength (UCS), the initial setting time, and the final setting time, were agreeable, with deviations from the expected UCS ranging from 0 to 38.12%, thus showing how the generated model is a reliable reference to estimate the UCS and setting time of low-calcium FA geopolymer paste for in situ applications.

Lee and colleagues assessed the preparation of potassium-based geopolymers (KGL) into leucite ceramics and tested their structure and mechanical properties [16]. By taking into consideration alkaline solution ratio, calcining temperature (T), and sintering, mixing, and curing time, they tested the prepared specimens to better understand the geopolymerization reactions and the characteristics of the KGL network. They found improved characteristics when the curing time increased due to the enhancement of the strong interaction between the matrix and the alkaline solution upon achieving adequate time to complete the geopolymerization process and forming a more stable three-dimensional structure. 

Kohout et al., prepared a metakaolinite-based geopolymer binder using calcined claystone as the main raw material and potassium as the alkaline activator, then proceed with their thermo-mechanical characterization [17]. By balancing the K/Al ratio, they studied the effects on the thermal and mechanical behavior of the prepared geopolymer, observing a decrease in the crystallization temperature of the new phases (leucite and kalsilite) as a function of a K/Al ratio increase. In the meantime, they observed a decrease in the compressive strength with increasing temperature, thus suggesting the use of low K/Al ratio geopolymers for thermal applications.

Morenov and coworkers carried out an interesting work to develop, improve, and study compositions of weighted drilling muds with low content of solids, on the basis of organic salts of alkali metals and polymers for the construction of wells prone to rock swelling and/or cavings, as well as drilling fluids for drilling-in the formation [18]. The developed drilling mud was characterized by a high inhibiting ability, allowing minimized mud-weighting by the natural solid phase. Among the benefits of the prepared composite, the authors highlighted the easy regulation of its properties by reducing the dispersion of drilled cuttings, as well as the absence of problems related to hydration and the swelling of active clay rocks and the stabilization of the unstable argillites prone to caving. 

Nafees et al., investigated the possible advantages related with the incorporation of silica fume (SF) as a partial substitution of cement in concrete, focusing on the development of modeling techniques in predicting the compressive strength of silica fume-based concrete [19]. In the designed model, the authors have identified six influential factors to be considered significant input parameters, such as cement, water, superplasticizer, silica fume, and fine and coarse aggregate. Individual and ensemble models of a decision tree and support vector machine allowed the authors to reach satisfactory results and high prediction accuracy. In particular, the last model showed an enhancement of 11 and 1.5% percent for decision tree and support vector machine compressive strength models, respectively. The authors concluded that cement and water are the governing parameters in developing compressive strength and, in addition, a cross-validation technique was used to avoid overfitting issues and confirm the generalized modeling output.

Reinforced and light-weight geopolymers can be employed in buildings, especially for walling, and in this framework Nguyen and coworkers exanimated the use of specific fillers for the metakaolin-based light-weight geopolymers, emphasizing properties such as high mechanical strengths, low thermal conductivity and density [20]. In their study, these authors pointed out polystyrene as the most suitable materials to be used as fillers, achieving five times lower thermal conductivity compared to cement concretes, which means five times lower heat loss by conduction, maintaining lightness with respect to the standard geopolymer composite. In the same study, the authors proposed Liapor and hollow ceramic microsphere as suitable fillers, which gave rise to better mechanical strengths of the obtained geopolymer, but worse results in terms of thermal conductivity compared to geopolymer polystyrene. 

The first of the two reviews contained in the Special Issue is by Ferdous et al., dealing with the purpose of reducing the amount of waste glass landfilled by transforming this waste into construction materials. It is well known that broken glass can be used in concrete either as a binder or aggregates. The outcomes of this knowledge are scattered, and it difficult to reach a firm conclusion about the effectiveness of waste glass in concrete, thus the authors critically reviewed its role and impact on microstructural and durability properties for both cement and geopolymer concrete [21].

In the second review, Siddika and colleagues strengthened the same topic, i.e., waste glass based geopolymers, focusing on the effect of the chemical and mineralogical composition, as well as glass particle and curing condition of concrete on the dissolution behavior and chemical reactivity of waste glass [22]. Considering the high pozzolanicity and lower alkali–silica reactivity of waste glass in concrete, these authors provided a review as regards the chemistry of waste glass in cement and geopolymer concrete.

## Data Availability

Not applicable.
